# Cetylpyridinium chloride mouth rinses alleviate experimental gingivitis by inhibiting dental plaque maturation

**DOI:** 10.1038/ijos.2016.18

**Published:** 2016-08-19

**Authors:** Fei Teng, Tao He, Shi Huang, Cun-Pei Bo, Zhen Li, Jin-Lan Chang, Ji-Quan Liu, Duane Charbonneau, Jian Xu, Rui Li, Jun-Qi Ling

**Affiliations:** 1 Department of Operative Dentistry and Endodontics, Guanghua School and Hospital of Stomatology, Sun Yat-sen University, Guangzhou, China; 2 Single-Cell Center and Shandong Key Laboratory of Energy Genetics, Qingdao Institute of Bioenergy and Bioprocess Technology, Chinese Academy of Sciences, Qingdao, China; 3 Department of Oral Care Clinical Operation, Procter & Gamble Mason Business Center, Cincinnati, USA; 4 Sino-Danish Center for Education and Research, University of Chinese Academy of Science, Beijing, China; 5 Department of Stomatology, Peking Union Medical College Hospital, Beijing, China; 6 Global Microbiology Capability Organization, Procter & Gamble Innovation Center, Beijing, China; 7 Global Microbiology Capability Organization, Procter & Gamble International Operations SA Singapore Branch, Singapore 138547, Singapore

**Keywords:** cetylpyridinium chloride, oral microbiota, oral rinse

## Abstract

Oral rinses containing chemotherapeutic agents, such as cetylpyridinium chloride (CPC), can alleviate plaque-induced gingival infections, but how oral microbiota respond to these treatments in human population remains poorly understood. *Via* a double-blinded, randomised controlled trial of 91 subjects, the impact of CPC-containing oral rinses on supragingival plaque was investigated in experimental gingivitis, where the subjects, after a 21-day period of dental prophylaxis to achieve healthy gingivae, received either CPC rinses or water for 21 days. Within-subject temporal dynamics of plaque microbiota and symptoms of gingivitis were profiled *via* 16S ribosomal DNA gene pyrosequencing and assessment with the Mazza gingival index. Cetylpyridinium chloride conferred gingival benefits, as progression of gingival inflammation resulting from a lack of dental hygiene was significantly slower in the mouth rinse group than in the water group due to inhibition of 17 gingivitis-enriched bacterial genera. Tracking of plaque α and β diversity revealed that CPC treatment prevents acquisition of new taxa that would otherwise accumulate but maintains the original biodiversity of healthy plaques. Furthermore, CPC rinses reduced the size, local connectivity and microbiota-wide connectivity of the bacterial correlation network, particularly for nodes representing gingivitis-enriched taxa. The findings of this study provide mechanistic insights into the impact of oral rinses on the progression and maturation of dental plaque in the natural human population.

## Introduction

Oral health is integral to general health and is essential for well-being.^1^ Gingivitis, a prevalent and reversible gum disease in human population, is characterised by inflammation of the gingivae in response to mature dental plaque biofilms. Persistent gingivitis may lead to chronic periodontitis in susceptible individuals, possibly resulting in irreversible destruction of periodontal tissue.^2^ Moreover, there is a link between gingivitis and cardiovascular risk.^3, 4^ Therefore, prevention and treatment of gingivitis are particularly significant to clinicians.

Daily tooth brushing is the most frequently recommended mechanical method for controlling supragingival plaque. It achieves this goal by physically interrupting plaque development and keeping plaque in an immature state. The efficacy of this endeavour, however, is often compromised by the presence of hard-to-reach areas, as well as inadequate skill, poor motivation and a lack of compliance.^5^ Consequently, the use of antimicrobial mouth rinses as a supplement to mechanical oral hygiene regimens is considered a valuable means of enhancing plaque control.^6, 7, 8, 9^ In particular, cetylpyridinium chloride (CPC), which carries a long history of safe and effective oral use, has frequently been employed as an antimicrobial ingredient to improve clinical efficacy.^10, 11^

Although the anti-bacterial efficacy of CPC mouth rinses has been well-documented *in vitro* and *in vivo*,^12, 13^ limitations are apparent. Most studies have focused on planktonic culture rather than dental plaque, which is one form of biofilm.^14^ Moreover, the impact of anti-bacterial ingredients on the not-yet-culturable components of oral microbiota is not clear; these bacteria may represent up to 50% of oral microorganisms.^15, 16^ This is particularly important as accumulating evidence has suggested that gingivitis is caused by a shift in microbiota structure that involves many bacteria instead of one or only a few.^12, 13, 17^ Furthermore, individual microbes may react to anti-bacterial agents differently; however, few studies have simultaneously profiled the plethora of bacterial inhabitants of plaque in any cohorts of significant size.^12, 18, 19, 20, 21^ Therefore, mechanistic dissection of and eventual rational improvements in the beneficial effects of CPC oral rinses necessitate probing the responses of plaque microbiota to CPC treatment in natural human population.

Here, a double-blinded, randomised controlled trial employing 91 patients was conducted. After a 3-week period of optimal oral hygiene that included dental prophylaxis, all subjects reached a state of healthy gingivae (that is, Baseline). The subjects were then randomised to receive either a CPC-containing rinse (41 subjects; CPC group) or a water-only preparation (50 subjects; control group, that is, experimental gingivitis group) for 21 days (Day 21). Within-subject temporal dynamics of plaque microbiota and clinical symptoms of gingivitis were profiled *via* 16S ribosomal RNA (rRNA) gene pyrosequencing and assessment *via* the Mazza gingival index (MGI). Our study provides mechanistic insights into the impact of CPC on the progression and maturation of dental plaque in an experimental gingivitis model.

## Materials and methods

A detailed description is provided in the Supplementary Materials. Experiments were conducted at Procter & Gamble (Beijing, China) Technology, Oral Care Department, with approval from the Procter & Gamble Beijing Technical Center (Beijing, China) Institutional Review Board and in accordance with the World Medical Association Declaration of Helsinki (1996 Amendment). The International Council for Harmonisation of Technical Requirements for Pharmaceuticals for Human Use (ICH) Guidelines for Good Clinical Practice were followed. Subjects were recruited from the Beijing area. The 91 total subjects first participated in an oral hygiene phase (Baseline). In this phase, each subject first received dental prophylaxis (supra- and subgingival prophylaxis) and tooth polishing and was then instructed to brush his/her teeth under supervision using a type of anti-cavity toothpaste (Crest Cavity Protection; Procter & Gamble, Guangzhou, China) for 3 min twice a day. This brushing regimen was followed for the next 21 days, resulting in the “Baseline” state of the gingivae. Then, the 91 subjects were randomly assigned into one of two groups: the CPC-treatment group (41 subjects) and the control group (50 subjects). Experimental gingivitis was induced by constraining subject oral hygiene practices, including brushing, flossing and dental prophylaxis, for the next 21 days. No toothpaste was used by any of the subjects during the study. To understand the impact of CPC oral rinses on gingivitis development and oral microbiomes, in the CPC-treatment group, subjects were instructed to return to the clinical site twice daily, at which time they would rinse with 20 mL of mouth rinse (Crest Pro-health Mouth Rinse; Procter & Gamble, Guangzhou, China) for 30 s. In the control group, subjects were instructed to return to the site twice daily, at which time they would rinse with 20 mL of water (purified drinking water made by Pepsi, Beijing, China) for 30 s.

Gingivitis and plaque examinations for each subject were performed at four time points: Baseline (Day 0) and Day 7, Day 14 and Day 21 of the experimental gingivitis (EG) phase. Their oral condition was assessed using the MGI, as in our previous study.^17, 22^ Bleeding on probing (BOP) frequency and mean MGI were then recorded for each subject.

Supragingival plaques from each of the individuals were collected after oral examination and analysed for microbial community structure. Genomic DNA was extracted from supragingival plaque at Baseline and at Day 21 of the experimental gingivitis (EG) phase. Barcoded 16S rRNA gene V1–V3 hypervariable regions (*Escherichia coli* positions 5–534) were sequenced on 454 Titanium, as we previously published.^22, 23^ In total, 182 plaque samples yielded 1 419 998 bacterial 16S rRNA gene sequences (containing the V1–V3 region) that passed stringent quality control,^24^ averaging 7 802 reads per sample (Supplementary Table S1). All sequences were deposited at Sequence Read Archive under Accession ID SRA063171. Pyrosequencing data of the subjects from the control group have been reported previously for the characterisation of gingivitis microbiomes.^17^

Sequences were analysed with the MOTHUR software package^25^ for preprocessing, taxonomic assignment and community-structure comparisons. The *X*^2^-statistic was used to test whether there was a difference in smoking status or gender between the CPC and control groups. A Wilcoxon rank-sum test was used to determine whether there was any age difference between the two groups. The clinical parameters (that is, BOP and the MGI), α diversity (that is, Shannon index and genus richness) and principal component 1 (PC1) values were compared between two groups *via* the Wilcoxon rank-sum test and within each of the groups using the Wilcoxon signed-rank test from Baseline to EG. Furthermore, the Wilcoxon rank-sum test was employed to test differences in within-subject changes in BOP and the MGI and intra-individual variations in PC1 (ΔPC1) between the CPC and control groups. Principal component analysis (PCA) was employed to test differences in plaque microbiota structure from Baseline to EG in the two groups. The correlation between microbial diversity (that is, Shannon index and PC1 values) and oral condition (that is, MGI) was evaluated *via* non-parametric Spearman's correlation. We tested the effect of CPC on microbial composition on both a cross-sectional and a longitudinal scale. (i) The difference in the relative abundance of each taxon was explored between the CPC and control groups at Day 21. (ii) From Baseline to EG, the extent of change in the relative abundance of each taxon within subjects between the CPC and control groups was assessed. Significant differences for each taxon between two groups were established by the Wilcoxon test and the false discovery rate (FDR) correction. Compositionality corrected by renormalization and permutation (CCREPE) (ref. 26) was employed to detect microbial interactions at the genus level. The data were exported and visualised using Cytoscape^27^ (http://www.cytoscape.org).

## Results

### Anti-gingivitis efficacy of CPC mouth rinses in an experimental gingivitis study

**Figure 1:**
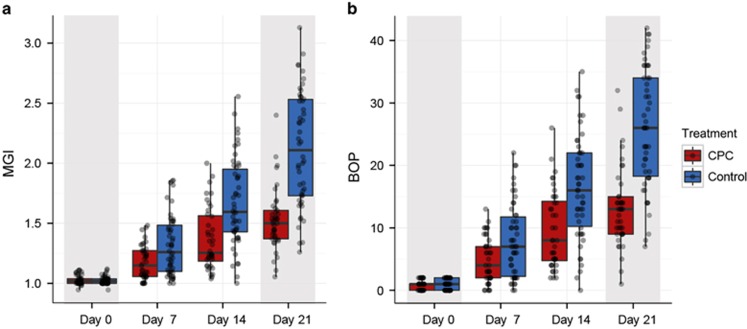
**Gingival health changes across experimental gingivitis study with or without CPC mouth rinse treatment.** Boxes represent the interquartile range (IQR), and the lines inside represent the median. Whiskers denote the lowest and highest values within 1.5 × IQR. Before Day 0, all subjects had received a rigorous oral hygiene regimen for 3 weeks, which resulted in greatly reduced MGI and BOP (median MGI and BOP were 1.02 and 1.00, respectively; “Baseline”) that corresponded to a healthy gum state. Then, the subjects further underwent an oral hygiene programme with and without CPC treatment for 3 weeks. In the control group, significantly increased BOP (mean 26.00±1.36) and MGI (mean 2.12±0.07) were observed compared with Baseline, whereas in the CPC group, slightly increased BOP (mean 13.17±0.99) and MGI (mean 1.53±0.04) were found. BOP, bleeding on probing; CPC, cetylpyridinium chloride; MGI, mazza gingival index.

Ninety one subjects were recruited for this study. All 91 subjects followed a rigorous oral hygiene regimen for 3 weeks before Baseline (Supplementary Methods). These subjects were randomly assigned into two groups that subsequently received water rinses (the experimental gingivitis group, called the control group here) or oral care mouth rinses (the CPC group) that featured regular usage of an oral hygiene product with CPC for 3 weeks (Day 21; Supplementary Methods and Figure 1). There was no significant difference between the CPC and control groups regarding either smoking status or gender based on a *X*^2^-analysis. The 91 human participants sampled consisted of 30 men and 61 women. The proportion of smokers was 24.4% for the CPC group and 26.0% for the control group (Supplementary Table S1). Ages ranged from 18 to 53 years and were not significantly different between the two groups (Supplementary Table S1).

At Baseline, between the two groups, the BOP (Supplementary Methods; Figure 1) values and the MGI (mean MGI of all teeth; Supplementary Methods) were not significantly different (*P*=0.84 for BOP and *P*=0.54 for MGI, Wilcoxon rank-sum test). In both groups, BOP values ranged from 0 to 2, while the MGI ranged from 0.94 to 1.12 at Baseline (Supplementary Table S1; Figure 1).

In the subsequent EG phase, at Day 21, the control group exhibited significantly increased BOP (mean 26.00±1.36) and MGI (mean 2.12±0.07) compared with Baseline; however, the CPC group displayed slightly increased BOP (mean 13.17±0.99) and MGI (mean 1.53±0.04). At Day 21 of the EG phase, despite the significantly increased BOP and MGI in both groups (*P*<0.01, Wilcoxon rank-sum test for both BOP and MGI), BOP and MGI were significantly lower in the CPC group than in the control group (*P*<0.01, Wilcoxon rank-sum test). Furthermore, the changes in BOP and MGI from Day 21 to Day 0 were calculated for each subject. BOP and MGI changes in the CPC group were significantly lower than those in the control group (*P*<0.01, Wilcoxon rank-sum test), suggesting the anti-gingivitis efficacy of CPC.

### CPC mouth rinses caused a shift in the structure of supragingival plaque during gingivitis development

**Figure 2:**
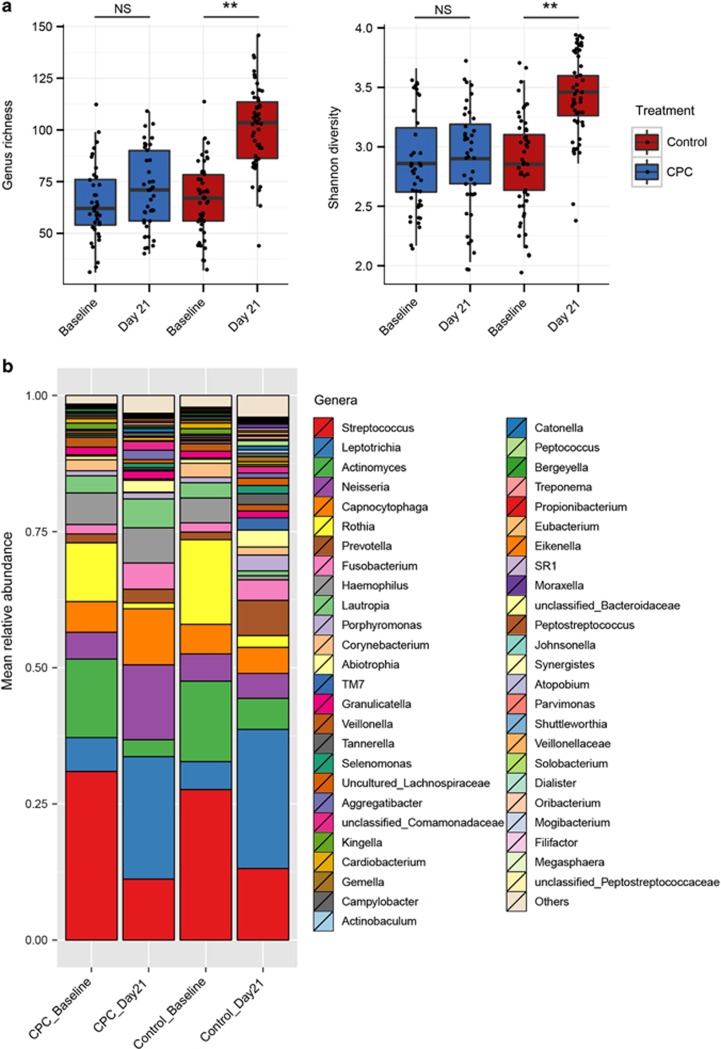
**Change in α diversity and bacterial taxonomic profiles in plaque microbiota with or without CPC treatment.** (**a**) The α diversity in the control group exhibited a significant increase from Baseline to Day 21, whereas that in the CPC group remained stable. These changes resulted in the profound differences in richness and in the Shannon index between the CPC and control groups at Day 21 underlying the distinct clinical symptoms between the two groups at Day 21. (**b**) Comparison of bacterial taxonomic profiles of the CPC and control groups from Baseline to Day 21 at the genus level. **P<0.01. CPC, cetylpyridinium chloride; NS, not significant.

To understand the changes in microbial diversity in the model of experimental gingivitis, the Shannon index and genus richness (α diversity) were calculated for each sample. The Shannon index and genus richness of the control group were significantly elevated from Baseline to Day 21 (Wilcoxon signed-rank test, *P*<0.01). However, no significant changes in α diversity were observed for the CPC group during the same period (Wilcoxon signed-rank test, *P*>0.05; Figure 2a). At Day 21, α diversity was profoundly higher in the control group than in the CPC group (Wilcoxon rank-sum test, *P*<0.01; Figure 2a). The increased microbial richness of the control group during gingivitis progression was driven by the higher detection rate of rare taxa (mean relative abundance <0.001) and the increased abundance of minority taxa (such as *Porphyromonas, Corynebacterium, Abiotrophia* and TM7) from Baseline to Day 21 (Figure 2b). However, such a trend was absent for the CPC-treatment group. These results indicate that CPC can affect the development of supragingival plaques that underlie gingivitis progression.

**Figure 3:**
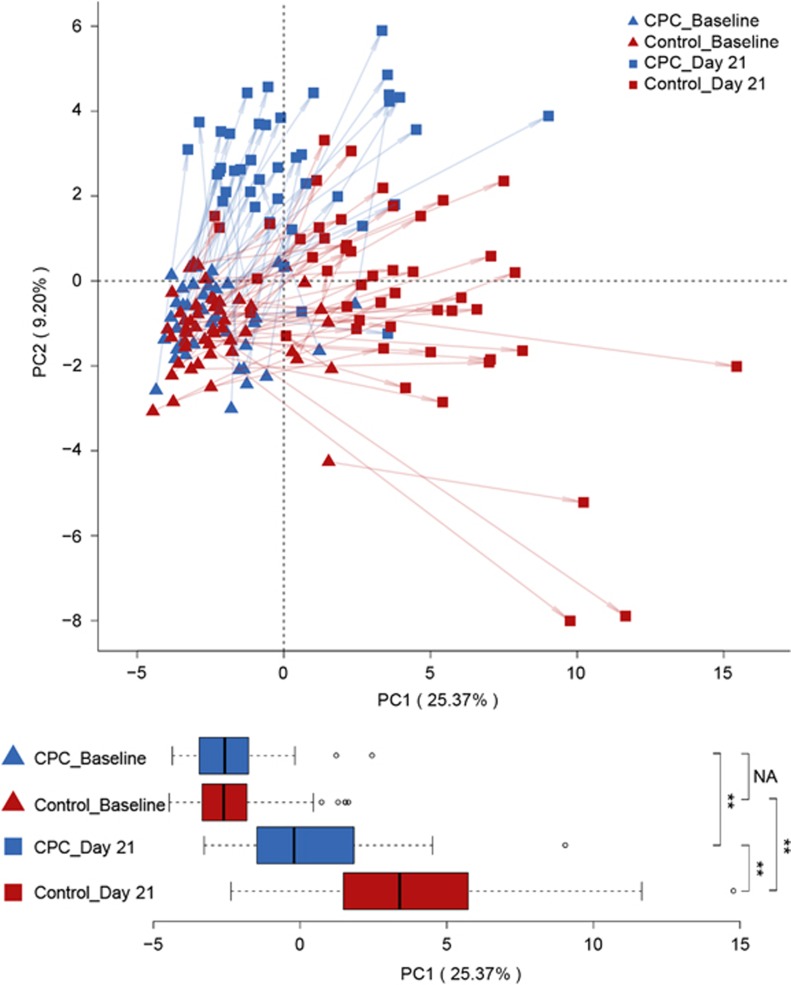
**Temporal changes in beta diversity of plaque microbiota with and without CPC treatment.** PC analysis revealed treatment as a dominant source of variation (each dot corresponds to plaque microbiota in the control group, while each triangle corresponds plaque microbiota in the CPC group, colour-coded by Baseline and Day 21). All samples were plotted on the first two PCs of the genus profile. Pre- and post-treatment plaque samples from the same individuals are connected by arrows. The first PC1 accounts for 25% of the variation in microbial changes among all samples. Shown below are boxplots of each sample's PC1 value grouped by treatments from Baseline to Day 21. Significant differences between the two groups were established using the Wilcoxon rank-sum test, while those within the two groups were established via the Wilcoxon signed-rank test (see bottom right legend). **P<0.01. PC, principal component; CPC, cetylpyridinium chloride; NA, not applicable.

To probe the changes in microbial community structure, PCA was performed for all subjects at Baseline and Day 21, and PC1 was employed to quantify the plaque microbiota response. At Day 21, the PC1 values of the CPC group were significantly lower than those of the control group (Wilcoxon rank-sum test, *P*<0.01; Figure 3). However, there was no such difference for the two groups regarding PC1 at Baseline. The degree of within-subject temporal variation was further compared between the two groups, as measured by the change in PC1 within-subject groups (ΔPC1). The intra-individual variation (ΔPC1) for the subjects in the CPC group was decidedly lower than that for the subjects in the control group (Wilcoxon rank-sum test, *P*<0.05). These results suggested structural segregation of the microbial community between the two groups after treatment. In addition, from Baseline to Day 21, ΔPC1 values were highly correlated with MGI changes (ΔMGI values) in the subject population (*P*<0.01, *ρ*=0.37, Spearman correlation), indicating that the observed changes in microbial community structure are clinically relevant.

### Seventeen gingivitis-associated bacterial genera are inhibited by CPC mouth rinses

**Figure 4:**
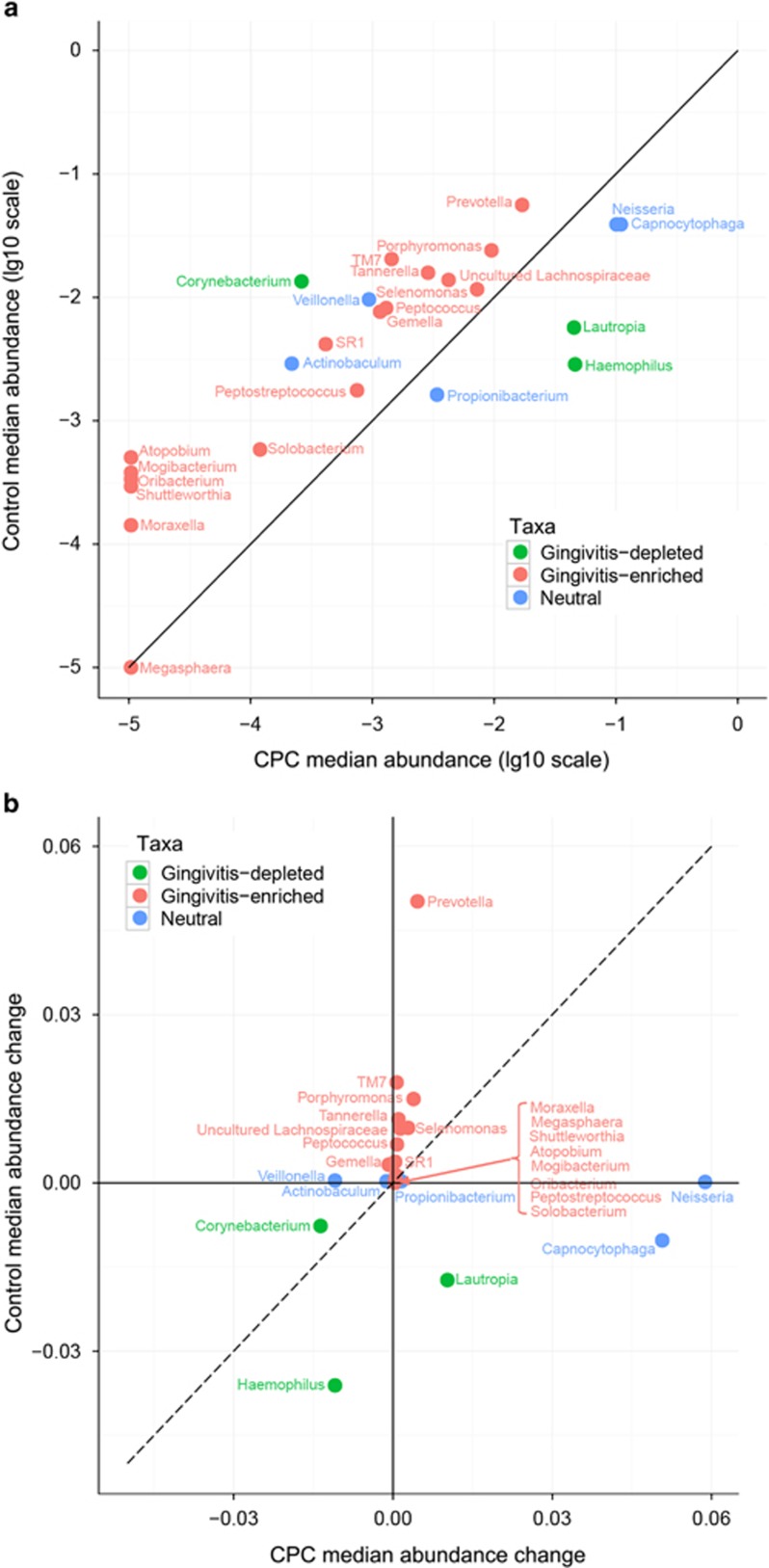
**Distinct temporal dynamics of 25 taxa from Baseline to Day 21 between the CPC and control groups.** (**a**) Between-treatment comparisons of bacterial abundance at each time point. The abundance change for each taxon was calculated as the abundance at Day 21 deducted from that at baseline. (**b**) The temporal changes in relative abundance for these 25 taxa were significantly (adjusted *P*<0.05, Wilcoxon rank-sum test) different between subjects in the CPC and control group. The dots were classified into three groups: red, gingivitis enriched; green, gingivitis depleted; blue, neutral. Bacterial classification was based on comparing the microbial profiles of the control group at Baseline and Day 21. CPC, cetylpyridinium chloride.

To investigate the impacts of CPC mouth rinses on individual bacterial taxa, the microbial profiles of the control group at Baseline and Day 21 were first compared, and then the oral bacterial genera were organised into three categories: 32 experimental gingivitis-enriched genera, 8 gingivitis-depleted genera and 10 neutral genera (Wilcoxon signed-rank test, *P*<0.01 FDR corrected; Supplementary Figure S1). Moreover, 25 bacterial genera were significantly modulated by CPC mouth rinses during experimental gingivitis development (Figure 4a and 4b). For each of these genera, the relative abundance at Day 21 and the temporal difference in abundance during the experiment (from Baseline to Day 21) were both significantly different between the control and CPC groups (Wilcoxon rank-sum test, *P*<0.01 FDR corrected). Among these 25 genera, 17 gingivitis-associated genera were significantly inhibited, including *Porphyromonas*, *Peptostreptococcus*, *Prevotella*, *Peptococcus*, *Selenomonas*, *Solobacterium*, *SR1*, *Tannerella*, TM7 genus, *Uncultured_Lachnospiraceae Atopobium*, *Gemella*, *Megasphaera*, *Mogibacterium*, *Moraxella*, *Oribacterium* and *Shuttleworthia* (Figures 4a and 4b). However, the relative abundances of gingivitis-depleted genera, such as *Haemophilus* and *Lautropia*, and neutral genera, such as *Neisseria, Capnocytophaga* and *Propioni-bacterium*, were significantly elevated for the subjects in the CPC group (Figure 4a and 4b).

### Impacts of CPC mouth rinses on bacterial interaction networks

**Figure 5:**
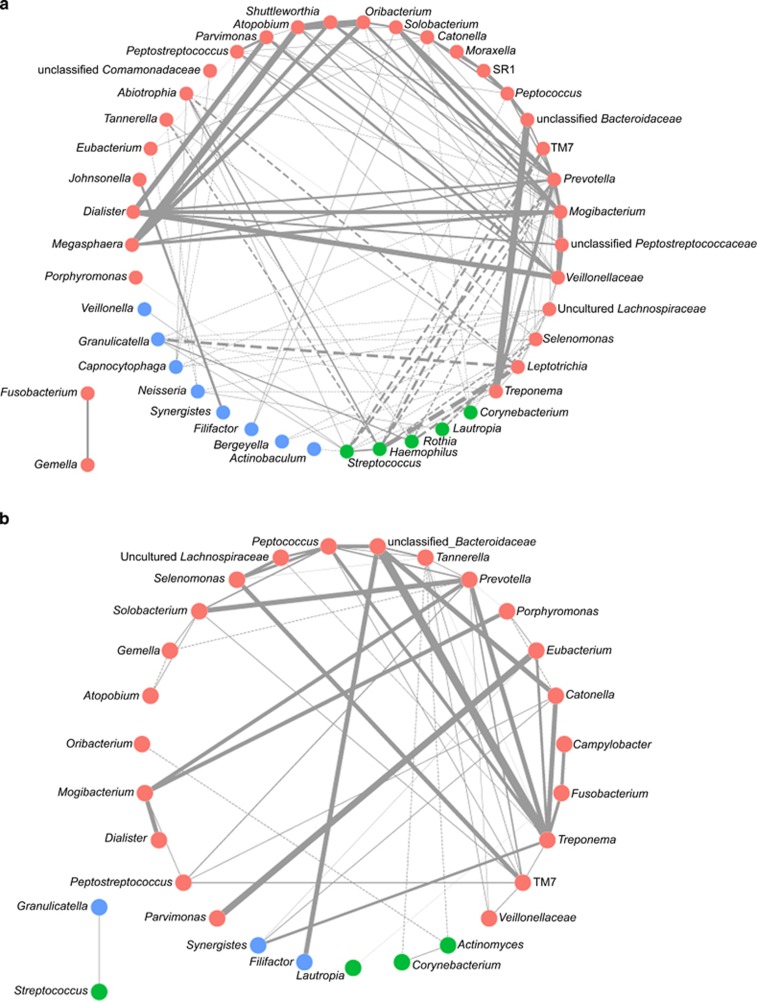
**Co-occurrence and co-exclusion relationships of oral bacteria in the two treatments.** Genus-level correlation networks were displayed for the control group and the CPC group at Day 21. Each node represents a bacterial genus, and each edge represents a significant co-occurrence (solid line)/co-exclusion (dotted line) relationship. Line thickness is proportional to the degree of correlation (Spearman's *ρ*). The nodes were classified into three groups: red, gingivitis enriched; green, gingivitis depleted; blue, neutral. CPC, cetylpyridinium chloride.

The presence of multiple bacterial taxa that respond to CPC treatment raised the possibility of CPC altering the intricate inter-microbe relationships that underlie the development of biofilms (that is, plaque). To test this hypothesis, the co-presence and co-exclusion relationships of oral bacteria were analysed for the subjects in the CPC group and the control group at Day 21 (Figure 5). Correlations were determined using CCREPE,^26^ and the Spearman similarity score for each taxon-taxon pair was summed across all samples in the responding groups.^26^

A number of observations were apparent (Figure 5). First of all, the size of the CPC group network was smaller than that of the control group, with the former composed of much fewer nodes (29) than the latter (43). Twenty seven nodes were shared between the two networks. Sixteen nodes were specific to the control group network, with nine of them being gingivitis-enriched taxa, such as *Leptotrichia* and *SR1*. However, only two nodes, including *Actinomyces* (gingivitis-depleted taxa) and *Campylobacter* (gingivitis-enriched taxa), were specific to the CPC group network. Thus, there were ninefold more gingivitis-enriched taxa among control-specific nodes than among CPC-specific nodes, suggesting that CPC may have disrupted certain interactions among gingivitis-enriched taxa during plaque development.

Moreover, the CPC group network consists of a lower number of inter-genera interactions than the control group network, as the former harbours 55 edges, which is in contrast to the 121 edges in the latter. For example, *Prevotella*, which is a gingivitis-enriched genus, was found to be connected to 20 other gingivitis-associated bacteria (16 gingivitis-enriched, 3 gingivitis-depleted and 1 neutral) in the control group network; however, it was linked to 10 other gingivitis-associated bacteria (10 gingivitis-enriched and no gingivitis-depleted) in the CPC group network. As *Prevotella* spp. have been shown, in past studies, to be implicated in endodontic,^28^ gingival^29^ and periodontal infections,^30^ such a difference in the connectivity of *Prevotella* spp. between the groups with and without CPC treatment further supports that CPC treatment reduces the connectivity of certain gingivitis-enriched taxa during plaque development.

Furthermore, the network-wide connectivity in the CPC network is lower as the average numbers of neighbours for each node in the CPC and control networks are 3.79 and 5.63, respectively. This suggests that CPC treatment inhibits formation and maturation of dental plaque by interfering with and reducing the synergetic interactions among bacterial taxa, which has been shown to be important for plaque development.^31, 32^

## Discussion

Dental plaque is a complex biofilm that undergoes maturation and, if not removed regularly, can lead to dental caries, gingivitis and periodontitis.^23, 30, 31, 33, 34^ By leveraging a model of experimental gingivitis in a 91-subject human cohort, mechanistic insights into the impact of CPC oral rinses on plaque composition and the clinical manifestation of gingivae were revealed.

First, CPC oral rinses are able to maintain the composition of plaque in a healthy immature state. This effect eventually prevents or slows down the progression of gingivitis. It is believed that health-associated plaque is generally immature,^35^ whereas gingivitis is associated with a more developed and complex microbial community.^13, 17^ A significant increase in the α-diversity of plaque microbiota was found for subjects in the control group from Baseline to Day 21, which is consistent with previous observations in other gingivitis cohorts.^30, 36, 37^ In contrast, during the same period, plaque α-diversity in the CPC-treated group remained stable, indicating that when starting with healthy gingivae CPC treatment prevents acquisition of new taxa that would otherwise accumulate but maintains the original biodiversity of healthy plaques that is associated with healthy gingivae.

Second, CPC oral rinses specifically inhibit increases in gingivitis-associated bacteria in supragingival biofilms during gingivitis development. Socransky *et al.*^38^ recognised that early plaque consists predominantly of gram positive organisms and that if left undisturbed, the plaque undergoes a process of maturation, resulting in predominantly gram-negative microbiota. In fact, 17 gingivitis-associated bacteria were significantly inhibited in the CPC-treated group compared with the placebo group. Among them, *Tannerella*, *Peptococcus*, *Selenomonas* and *Prevotella* are Gram-negative obligate anaerobic microorganisms that are embedded in matrices of polymers in thick and deep dental biofilms.^31^ Studies based on traditional methods, such as viable counting and confocal microscopy, also supported the anti-bacterial and anti-biofilm effectiveness of CPC on certain culturable pathogenic bacteria (such as *Porphyromonas gingivalis*, *Prevotella intermedia* and *Prevotella nigrescens*), anaerobic bacterial communities and even oral biofilms.^7, 20, 39, 40^ This suggests that CPC can diffuse into oral biofilms and exert anti-bacterial effects *in vivo*.^41^ Notably, oral bacteria that were difficult to grow in culture or were not yet cultured, such as TM7, SR1 and Uncultured_*Lachnospiraceae*, were also inhibited by CPC mouth rinses, suggesting the importance of culture-independent approaches in unveiling a global landscape of microbial responses in plaque. To the best of our knowledge, this is the first study evaluating the preventative efficacy of CPC in experimental gingivitis, as well as its impact on oral microbiome changes, by 16S rRNA gene sequencing. The identified microbiota changes are consistent with overall microbiota changes after long- and short-term periodontal therapy. For example, *Tannerella* and *Porphyromonas*, which had been classified as part of the red complex for periodontal disease, were found to be significantly inhibited by CPC in our study and also shown to be reduced in other periodontal therapies.^38, 42, 43^

Third, CPC oral rinses appear to disrupt or reduce the ecological interactions that underlie the development of plaque during gingivitis development. The co-adhesion of different oral bacteria is a critical step for building physical networks. Our results suggested that the size, local connectivity and microbiota-wide connectivity of bacterial correlation networks were reduced by CPC treatment, particularly for those nodes representing bacterial taxa that are positively associated with gingivitis. For example, a negative correlation between *Streptococcus* and *Prevotella* was observed in the control samples of this study at Day 21 (which was consistent with an inverse correlation between the two bacterial taxa found in periodontal pocket microbiota in periodontitis patients).^44^ However, such a connection was disrupted or lost in the CPC groups. It is notable, however, that various CPC-containing formulations can have distinct pellicle surface properties and thus may exert different impacts on such bacterial interactions (the Crest Pro-health Mouth Rinse used herein features high bioavailability of CPC, which yields more hydrophobic pellicles on the tooth surface and interferes with bacterial co-adhesion).^45^ Moreover, tracking the dynamics of such bacterial correlation networks along short- or long-term treatment with CPC oral rinses may help to reconstruct the successive patterns of each individual taxon and thus reveal the causal relationships among interacting plaque microbes in response to CPC. Comparisons of such treatment-induced response patterns of plaque microbiota among different types of oral rinses may serve as a valuable venue for the mechanism-based evaluation of oral care products and for guiding rational development of oral care regimens.

Therefore, a CPC-containing mouth rinse, when used as the only oral hygiene regimen, provides a significant benefit in reducing gingival inflammation by disturbing the succession of dental plaque maturation (that is, gingivitis-associated microbiota) and balancing the diversity and composition of the oral microbiota (that is, health-associated microbiota). Taken together, this fresh view of the antimicrobial effects of CPC from a micro-ecological perspective provides new opportunities for developing new strategies for prevention and control of plaque-mediated diseases, such as gingivitis and periodontitis.

## Notes

The co-authors Rui Li, Tao He, Jin-Lan Chang, Ji-Quan Liu and Duane Charbonneau contributed to and/or conducted this study while being employed by the Procter & Gamble Company. These co-authors may own stock in the company, but direct financial gain or loss from the publication of this manuscript is not anticipated. Under a requirement of employment obligations of the Procter & Gamble Company to maintain confidentiality, these co-authors cannot declare patent activity. Financial support from The Procter & Gamble Company, Cincinnati, OH was used to design and conduct part of the study.
