# Supplementary figures and images for: Cetylpyridinium chloride mouth rinses alleviate experimental gingivitis by inhibiting dental plaque maturation
Source: Int J Oral Sci. 2016 Aug 19;8(3):182–90. doi: 10.1038/ijos.2016.18 (PMC5113089; doi:10.1038/ijos.2016.18)

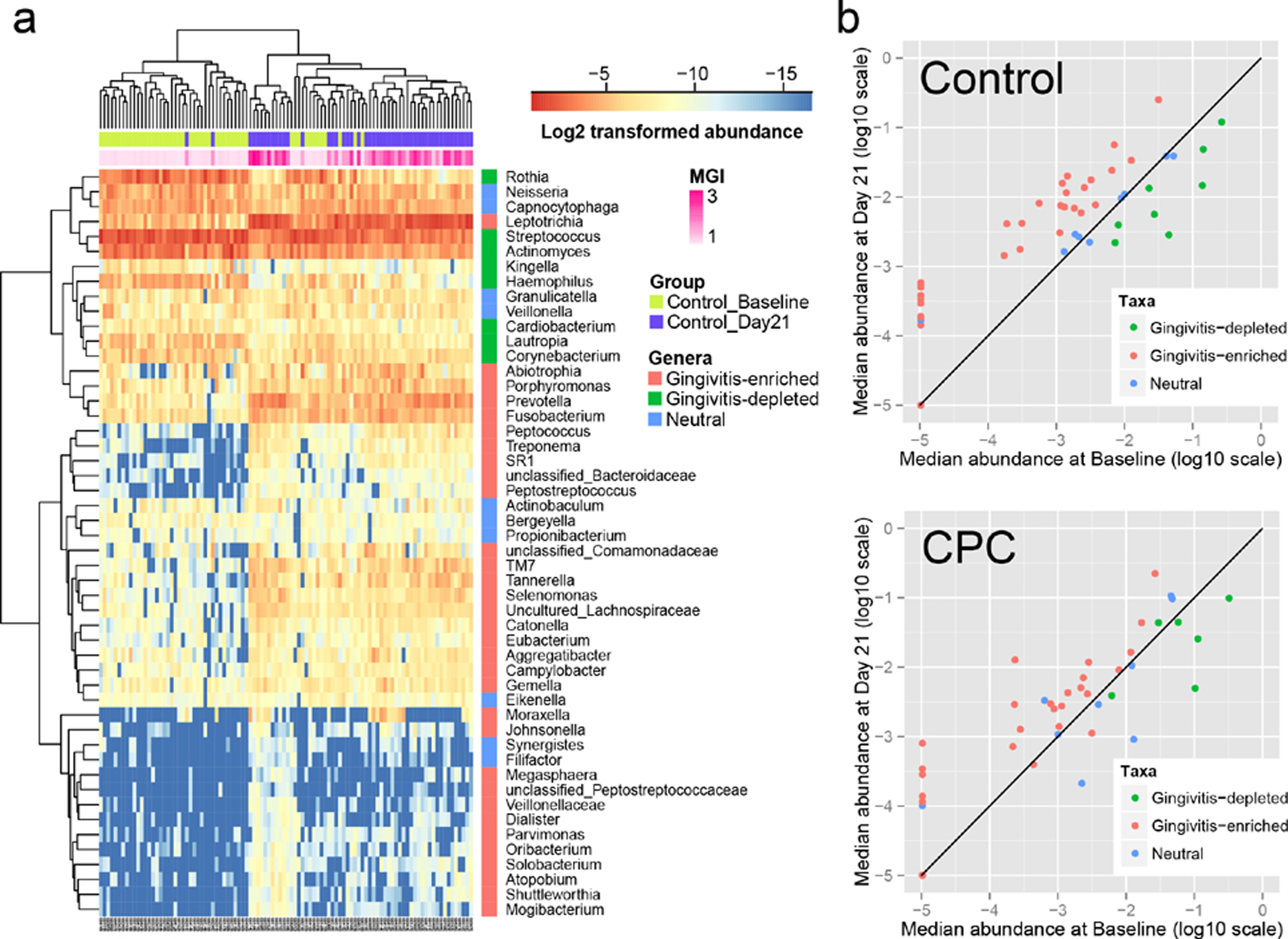

Supplement: Supplementary Figure 1 [file ijos201618x1.tif]
