# Supplementary material for: Cetylpyridinium chloride mouth rinses alleviate experimental gingivitis by inhibiting dental plaque maturation
Source: Int J Oral Sci. 2016 Aug 19;8(3):182–90. doi: 10.1038/ijos.2016.18 (PMC5113089; doi:10.1038/ijos.2016.18)
Supplement: Supplementary Information [file ijos201618x2.docx]

**Supplementary Tables and Figures**

**Table S1. Clinical parameters of all 182 samples.**

| **Sample ID** | **Subject Group** | **Time-point** | **Host ID** | **MGI** | **Bleeding** | **Age** | **Sex** | **Smoking** |
| --- | --- | --- | --- | --- | --- | --- | --- | --- |
| G9051B | CPC | Baseline | 9051 | 1 | 0 | 48 | F | No |
| G9130B | CPC | Baseline | 9130 | 1 | 0 | 43 | F | No |
| G9154B | CPC | Baseline | 9154 | 1.1 | 2 | 39 | F | No |
| G9157B | CPC | Baseline | 9157 | 1.09 | 2 | 31 | F | No |
| G9159B | CPC | Baseline | 9159 | 1 | 0 | 24 | F | No |
| G9163B | CPC | Baseline | 9163 | 1.04 | 1 | 23 | F | No |
| G9164B | CPC | Baseline | 9164 | 1.11 | 2 | 47 | F | No |
| G9180B | CPC | Baseline | 9180 | 1 | 0 | 39 | M | No |
| G9181B | CPC | Baseline | 9181 | 1 | 0 | 33 | F | No |
| G9192B | CPC | Baseline | 9192 | 1 | 0 | 27 | F | No |
| G9193B | CPC | Baseline | 9193 | 1.02 | 1 | 25 | M | Yes |
| G9197B | CPC | Baseline | 9197 | 1 | 0 | 37 | F | No |
| G9204B | CPC | Baseline | 9204 | 1.11 | 2 | 53 | F | No |
| G9210B | CPC | Baseline | 9210 | 1.04 | 1 | 30 | F | No |
| G9212B | CPC | Baseline | 9212 | 1.02 | 1 | 27 | F | No |
| G9218B | CPC | Baseline | 9218 | 0.95 | 0 | 25 | F | No |
| G9229B | CPC | Baseline | 9229 | 1 | 0 | 23 | M | Yes |
| G9230B | CPC | Baseline | 9230 | 1.07 | 2 | 33 | F | No |
| G9269B | CPC | Baseline | 9269 | 0.98 | 0 | 26 | F | No |
| G9287B | CPC | Baseline | 9287 | 1.07 | 2 | 29 | M | Yes |
| G9316B | CPC | Baseline | 9316 | 1 | 0 | 34 | F | No |
| G9317B | CPC | Baseline | 9317 | 1.07 | 2 | 25 | M | Yes |
| G9323B | CPC | Baseline | 9323 | 1 | 0 | 37 | M | Yes |
| G9329B | CPC | Baseline | 9329 | 0.98 | 0 | 24 | M | Yes |
| G9331B | CPC | Baseline | 9331 | 1.04 | 2 | 38 | F | No |
| G9333B | CPC | Baseline | 9333 | 1 | 0 | 42 | F | No |
| G9336B | CPC | Baseline | 9336 | 1.02 | 1 | 37 | M | Yes |
| G9348B | CPC | Baseline | 9348 | 1.02 | 1 | 39 | F | No |
| G9355B | CPC | Baseline | 9355 | 1.04 | 1 | 26 | M | Yes |
| G9368B | CPC | Baseline | 9368 | 1.02 | 1 | 24 | M | Yes |
| G9369B | CPC | Baseline | 9369 | 1.02 | 1 | 19 | M | No |
| G9375B | CPC | Baseline | 9375 | 1 | 0 | 33 | F | No |
| G9401B | CPC | Baseline | 9401 | 1 | 0 | 45 | F | No |
| G9402B | CPC | Baseline | 9402 | 1.11 | 2 | 29 | M | Yes |
| G9411B | CPC | Baseline | 9411 | 1.09 | 2 | 29 | M | No |
| G9429B | CPC | Baseline | 9429 | 1.06 | 1 | 28 | F | No |
| G9432B | CPC | Baseline | 9432 | 1 | 0 | 36 | F | No |
| G9434B | CPC | Baseline | 9434 | 1.02 | 1 | 26 | F | No |
| G9438B | CPC | Baseline | 9438 | 1 | 0 | 24 | F | No |
| G9456B | CPC | Baseline | 9456 | 1.04 | 1 | 35 | F | No |
| G9461B | CPC | Baseline | 9461 | 1.02 | 1 | 31 | F | No |
| G9066B | WATER | Baseline | 9066 | 1 | 0 | 32 | F | No |
| G9078B | WATER | Baseline | 9078 | 1 | 0 | 22 | F | No |
| G9079B | WATER | Baseline | 9079 | 1.04 | 2 | 26 | M | No |
| G9116B | WATER | Baseline | 9116 | 1 | 0 | 46 | F | Yes |
| G9126B | WATER | Baseline | 9126 | 1 | 0 | 21 | M | Yes |
| G9138B | WATER | Baseline | 9138 | 1.04 | 2 | 23 | M | Yes |
| G9147B | WATER | Baseline | 9147 | 1.04 | 2 | 28 | M | No |
| G9148B | WATER | Baseline | 9148 | 1.07 | 2 | 41 | F | No |
| G9151B | WATER | Baseline | 9151 | 1.02 | 1 | 31 | F | No |
| G9153B | WATER | Baseline | 9153 | 1.02 | 1 | 31 | F | No |
| G9155B | WATER | Baseline | 9155 | 1 | 0 | 42 | M | Yes |
| G9158B | WATER | Baseline | 9158 | 1.07 | 2 | 38 | F | No |
| G9168B | WATER | Baseline | 9168 | 1 | 0 | 29 | F | No |
| G9170B | WATER | Baseline | 9170 | 1 | 0 | 38 | F | No |
| G9174B | WATER | Baseline | 9174 | 1.05 | 2 | 36 | F | No |
| G9175B | WATER | Baseline | 9175 | 1.02 | 1 | 35 | F | No |
| G9182B | WATER | Baseline | 9182 | 0.94 | 0 | 27 | F | No |
| G9183B | WATER | Baseline | 9183 | 1.02 | 1 | 26 | M | Yes |
| G9196B | WATER | Baseline | 9196 | 1.12 | 2 | 50 | F | No |
| G9202B | WATER | Baseline | 9202 | 1 | 0 | 52 | F | No |
| G9203B | WATER | Baseline | 9203 | 1 | 0 | 25 | M | Yes |
| G9222B | WATER | Baseline | 9222 | 1 | 0 | 41 | F | No |
| G9226B | WATER | Baseline | 9226 | 1.04 | 2 | 29 | F | No |
| G9228B | WATER | Baseline | 9228 | 1.04 | 1 | 34 | F | No |
| G9251B | WATER | Baseline | 9251 | 1 | 0 | 36 | F | No |
| G9296B | WATER | Baseline | 9296 | 1.11 | 2 | 37 | M | No |
| G9305B | WATER | Baseline | 9305 | 1 | 0 | 24 | M | Yes |
| G9307B | WATER | Baseline | 9307 | 1.04 | 2 | 34 | F | No |
| G9309B | WATER | Baseline | 9309 | 1.02 | 1 | 23 | M | Yes |
| G9318B | WATER | Baseline | 9318 | 1 | 0 | 29 | F | No |
| G9320B | WATER | Baseline | 9320 | 1.02 | 1 | 36 | M | Yes |
| G9325B | WATER | Baseline | 9325 | 1 | 0 | 32 | F | No |
| G9364B | WATER | Baseline | 9364 | 1 | 0 | 34 | M | Yes |
| G9378B | WATER | Baseline | 9378 | 1.04 | 1 | 35 | F | No |
| G9383B | WATER | Baseline | 9383 | 1 | 0 | 48 | F | No |
| G9389B | WATER | Baseline | 9389 | 1.02 | 1 | 21 | F | No |
| G9405B | WATER | Baseline | 9405 | 1 | 0 | 37 | F | No |
| G9406B | WATER | Baseline | 9406 | 1.04 | 2 | 18 | M | Yes |
| G9412B | WATER | Baseline | 9412 | 1 | 0 | 39 | M | Yes |
| G9414B | WATER | Baseline | 9414 | 1.09 | 2 | 26 | M | No |
| G9415B | WATER | Baseline | 9415 | 1.02 | 1 | 22 | F | No |
| G9416B | WATER | Baseline | 9416 | 1 | 0 | 30 | F | No |
| G9419B | WATER | Baseline | 9419 | 1 | 0 | 25 | M | Yes |
| G9436B | WATER | Baseline | 9436 | 1.04 | 2 | 31 | F | No |
| G9439B | WATER | Baseline | 9439 | 1 | 0 | 27 | F | No |
| G9441B | WATER | Baseline | 9441 | 1.06 | 1 | 43 | F | No |
| G9445B | WATER | Baseline | 9445 | 1.05 | 2 | 27 | M | No |
| G9446B | WATER | Baseline | 9446 | 1 | 0 | 27 | F | No |
| G9457B | WATER | Baseline | 9457 | 1.05 | 2 | 24 | F | No |
| G9466B | WATER | Baseline | 9466 | 1.02 | 1 | 30 | F | No |
| G9051E | CPC | EG | 9051 | 1.43 | 9 | 48 | F | No |
| G9130E | CPC | EG | 9130 | 1.48 | 13 | 43 | F | No |
| G9154E | CPC | EG | 9154 | 1.5 | 13 | 39 | F | No |
| G9157E | CPC | EG | 9157 | 1.64 | 14 | 31 | F | No |
| G9159E | CPC | EG | 9159 | 1.5 | 14 | 24 | F | No |
| G9163E | CPC | EG | 9163 | 1.18 | 5 | 23 | F | No |
| G9164E | CPC | EG | 9164 | 1.63 | 15 | 47 | F | No |
| G9180E | CPC | EG | 9180 | 1.59 | 14 | 39 | M | No |
| G9181E | CPC | EG | 9181 | 1.54 | 14 | 33 | F | No |
| G9192E | CPC | EG | 9192 | 1.11 | 3 | 27 | F | No |
| G9193E | CPC | EG | 9193 | 1.82 | 20 | 25 | M | Yes |
| G9197E | CPC | EG | 9197 | 1.38 | 7 | 37 | F | No |
| G9204E | CPC | EG | 9204 | 1.41 | 10 | 53 | F | No |
| G9210E | CPC | EG | 9210 | 1.4 | 11 | 30 | F | No |
| G9212E | CPC | EG | 9212 | 1.21 | 7 | 27 | F | No |
| G9218E | CPC | EG | 9218 | 1.36 | 11 | 25 | F | No |
| G9229E | CPC | EG | 9229 | 1.36 | 10 | 23 | M | Yes |
| G9230E | CPC | EG | 9230 | 1.37 | 9 | 33 | F | No |
| G9269E | CPC | EG | 9269 | 1.25 | 7 | 26 | F | No |
| G9287E | CPC | EG | 9287 | 1.05 | 1 | 29 | M | Yes |
| G9316E | CPC | EG | 9316 | 1.55 | 12 | 34 | F | No |
| G9317E | CPC | EG | 9317 | 1.35 | 10 | 25 | M | Yes |
| G9323E | CPC | EG | 9323 | 1.52 | 13 | 37 | M | Yes |
| G9329E | CPC | EG | 9329 | 1.68 | 20 | 24 | M | Yes |
| G9331E | CPC | EG | 9331 | 1.57 | 17 | 38 | F | No |
| G9333E | CPC | EG | 9333 | 1.69 | 15 | 42 | F | No |
| G9336E | CPC | EG | 9336 | 1.98 | 24 | 37 | M | Yes |
| G9348E | CPC | EG | 9348 | 1.44 | 9 | 39 | F | No |
| G9355E | CPC | EG | 9355 | 1.34 | 9 | 26 | M | Yes |
| G9368E | CPC | EG | 9368 | 1.54 | 13 | 24 | M | Yes |
| G9369E | CPC | EG | 9369 | 2.05 | 29 | 19 | M | No |
| G9375E | CPC | EG | 9375 | 1.54 | 18 | 33 | F | No |
| G9401E | CPC | EG | 9401 | 1.33 | 8 | 45 | F | No |
| G9402E | CPC | EG | 9402 | 1.45 | 10 | 29 | M | Yes |
| G9411E | CPC | EG | 9411 | 1.79 | 21 | 29 | M | No |
| G9429E | CPC | EG | 9429 | 1.54 | 14 | 28 | F | No |
| G9432E | CPC | EG | 9432 | 2.4 | 32 | 36 | F | No |
| G9434E | CPC | EG | 9434 | 1.96 | 23 | 26 | F | No |
| G9438E | CPC | EG | 9438 | 1.57 | 14 | 24 | F | No |
| G9456E | CPC | EG | 9456 | 1.44 | 9 | 35 | F | No |
| G9461E | CPC | EG | 9461 | 1.61 | 13 | 31 | F | No |
| G9066E | WATER | EG | 9066 | 1.93 | 26 | 32 | F | No |
| G9078E | WATER | EG | 9078 | 1.55 | 14 | 22 | F | No |
| G9079E | WATER | EG | 9079 | 1.52 | 16 | 26 | M | No |
| G9116E | WATER | EG | 9116 | 1.54 | 12 | 46 | F | Yes |
| G9126E | WATER | EG | 9126 | 1.34 | 9 | 21 | M | Yes |
| G9138E | WATER | EG | 9138 | 2.02 | 23 | 23 | M | Yes |
| G9147E | WATER | EG | 9147 | 2.34 | 29 | 28 | M | No |
| G9148E | WATER | EG | 9148 | 3.13 | 41 | 41 | F | No |
| G9151E | WATER | EG | 9151 | 2.05 | 28 | 31 | F | No |
| G9153E | WATER | EG | 9153 | 2.2 | 26 | 31 | F | No |
| G9155E | WATER | EG | 9155 | 1.74 | 17 | 42 | M | Yes |
| G9158E | WATER | EG | 9158 | 2.7 | 38 | 38 | F | No |
| G9168E | WATER | EG | 9168 | 2.55 | 36 | 29 | F | No |
| G9170E | WATER | EG | 9170 | 2.18 | 27 | 38 | F | No |
| G9174E | WATER | EG | 9174 | 2.63 | 39 | 36 | F | No |
| G9175E | WATER | EG | 9175 | 2.91 | 42 | 35 | F | No |
| G9182E | WATER | EG | 9182 | 2.81 | 36 | 27 | F | No |
| G9183E | WATER | EG | 9183 | 1.77 | 19 | 26 | M | Yes |
| G9196E | WATER | EG | 9196 | 2.26 | 23 | 50 | F | No |
| G9202E | WATER | EG | 9202 | 2.55 | 36 | 52 | F | No |
| G9203E | WATER | EG | 9203 | 1.73 | 18 | 25 | M | Yes |
| G9222E | WATER | EG | 9222 | 1.65 | 14 | 41 | F | No |
| G9226E | WATER | EG | 9226 | 2.45 | 31 | 29 | F | No |
| G9228E | WATER | EG | 9228 | 1.83 | 23 | 34 | F | No |
| G9251E | WATER | EG | 9251 | 1.73 | 18 | 36 | F | No |
| G9296E | WATER | EG | 9296 | 1.69 | 14 | 37 | M | No |
| G9305E | WATER | EG | 9305 | 2.41 | 31 | 24 | M | Yes |
| G9307E | WATER | EG | 9307 | 2.33 | 32 | 34 | F | No |
| G9309E | WATER | EG | 9309 | 2.82 | 40 | 23 | M | Yes |
| G9318E | WATER | EG | 9318 | 2 | 21 | 29 | F | No |
| G9320E | WATER | EG | 9320 | 2.77 | 37 | 36 | M | Yes |
| G9325E | WATER | EG | 9325 | 2.52 | 36 | 32 | F | No |
| G9364E | WATER | EG | 9364 | 1.46 | 18 | 34 | M | Yes |
| G9378E | WATER | EG | 9378 | 2.16 | 26 | 35 | F | No |
| G9383E | WATER | EG | 9383 | 2.66 | 41 | 48 | F | No |
| G9389E | WATER | EG | 9389 | 1.82 | 20 | 21 | F | No |
| G9405E | WATER | EG | 9405 | 1.33 | 8 | 37 | F | No |
| G9406E | WATER | EG | 9406 | 1.26 | 7 | 18 | M | Yes |
| G9412E | WATER | EG | 9412 | 2.38 | 34 | 39 | M | Yes |
| G9414E | WATER | EG | 9414 | 1.96 | 23 | 26 | M | No |
| G9415E | WATER | EG | 9415 | 1.64 | 22 | 22 | F | No |
| G9416E | WATER | EG | 9416 | 2.54 | 34 | 30 | F | No |
| G9419E | WATER | EG | 9419 | 2.36 | 30 | 25 | M | Yes |
| G9436E | WATER | EG | 9436 | 2.57 | 34 | 31 | F | No |
| G9439E | WATER | EG | 9439 | 1.68 | 19 | 27 | F | No |
| G9441E | WATER | EG | 9441 | 2.52 | 33 | 43 | F | No |
| G9445E | WATER | EG | 9445 | 1.98 | 26 | 27 | M | No |
| G9446E | WATER | EG | 9446 | 1.66 | 16 | 27 | F | No |
| G9457E | WATER | EG | 9457 | 2.61 | 36 | 24 | F | No |
| G9466E | WATER | EG | 9466 | 1.79 | 21 | 30 | F | No |

**Figure S1. Temporal patterns of the most abundant 50 taxa from Baseline to Day 21 in the CPC and control subject groups.** (**a**) The heat map shows the log2-transformed abundance of the 50 most abundant genera in each sample collected in the control group. The genera were classified as gingivitis-enriched, gingivitis-depleted or neutral (adjusted *P*<0.05, Wilcoxon rank-sum test). (**b**) The median temporal changes in these 50 taxa within the control and CPC groups are shown as the scatter plot.
