# Supplementary material for: Cetylpyridinium chloride mouth rinses alleviate experimental gingivitis by inhibiting dental plaque maturation
Source: Int J Oral Sci. 2016 Aug 19;8(3):182–90. doi: 10.1038/ijos.2016.18 (PMC5113089; doi:10.1038/ijos.2016.18)
Supplement: Supplementary Information [file ijos201618x3.docx]

**Supplementary Materials**

**Materials and Methods**

***Study design*** The experimental model of gingivitis was established as a non-invasive model in humans for understanding the clinical and antimicrobial efficacy of cetylpyridinium chloride (CPC) on gingivitis ^1-4^. Experiments were conducted at Procter & Gamble (Beijing) Technology Co., Ltd. Oral Care Department, with approval from the P&G Beijing Technical Center (China) Institutional Review Board and in accordance with the World Medical Association Declaration of Helsinki (1996 amendment). ICH Guidelines for Good Clinical Practice (GCPs) were followed.

Ninety-one subjects were recruited from the Beijing area. They participated in Oral Hygiene Phase (Baseline), and then randomized to the CPC-treatment (41 subjects, i.e., the CPC group) group and the water-treatment group (50 subjects, i.e., the control group) in Experimental Phase (Day 21, EG) for 21 days. Except the 41 samples in the CPC group at Day 21, the other samples were related to our other studies ^5^. Voluntary informed consent was provided. Individuals meeting the following criteria were included: be at least 18 years of age; possess a minimum of 12 natural anterior teeth; have at least 5 bleeding sites as measured by Mazza Gingival Index (MGI) at initial visit at Day -21; be in good general health as determined by the investigator/designee based on a review of the medical history/update for participation in the study. Individuals meeting the following criteria were NOT included: periodontitis, i.e. presence of four teeth with ≥5 mm pockets in two quadrants; severe periodontal disease, as characterized by purulent exudates, generalized mobility, and/or severe recession; use of antibiotics any time during the study; any condition which requires antibiotic premedication for the administration of a dental prophylaxis; self-reported pregnancy or intent to become pregnant during the course of the study and nursing females; fixed facial orthodontic appliances; atypical discoloration or pigmentation in the gingival tissue; atypical discoloration or pigmentation in the gingival tissue; any diseases or conditions that could be expected to interfere with the subject safely completing the study. All of the subjects completed the experimental protocol between Jan 26^th^ and April 9^th^, 2011.

Study included two phases. Phase I, Oral Hygiene Phase (Day -21 to Baseline): Gingivitis examinations using Mazza Gingival Index (MGI) were conducted at -21, -14, -7 and 0 days (Baseline). After receiving a dental prophylaxis (super and sub gingival prophylaxis) and tooth polishing, each subject was instructed to return to the site twice daily at which time they brushed under supervision using Mei Li Liang Jie manual toothbrush (Crest, Made in China) for three minutes with a currently marketed anti-cavity dentifrice without any marked anti-microbial actives and then use the floss to clean the dental interproximal area. This brushing regimen was followed for the next 21 days while recording MGI for each subject each visit. During the Oral Hygiene Phase, subjects received up to three dental prophylaxes if their bleeding sites are more than 1.

Phase II, Experimental Gingivitis Phase (Baseline to Day 21): ninety-one subjects were randomized to to receive CPC (41 subjects, the CPC group) and water treatment (50 subjects, the control group) for 21 days. There would be at least 4 hrs in between the twice product usage instruction, no eating/drinking for 30min post mouth rinsing. During this phase, the subjects did not have any other oral hygiene practice including brushing, mouth rinsing with any products, flossing and dental prophylaxis. The subjects also received a gingivitis exam at days 7, 14 and 21 of the Experimental Gingivitis Phase.

In the CPC group, the subjects were instructed to return to the site twice daily at which time they would rinse with the 20ml mouth rinse (Crest® Pro health mouth rinse) for 30 seconds. However, in the control group, the subjects were instructed to return to the site twice daily at which time they will rinse with 20ml water (Purified Drinking Water made by Pepsi Company) for 30 seconds.

Gingivitis was assessed using MGI (Mazza Gingival Index) as in our previous study ^6^. BOP (Bleeding on Probing) frequency and mean MGI were recorded for each subject. MGI measures both the signs of inflammation as well as the degree of the severity of bleeding. Specifically, probing was performed by a dentist on the mesiobuccal and the distolingual of each tooth, for a maximum of 56 sites. BOP (Bleeding on Probing) frequency and mean MGI were recorded for each subject. Scores range from 0-5, with 0 assigned for normal appearing and healthy gingiva up to a score of 5 for spontaneous bleeding (without provocation). MGI of all subjects were measured by the same well-trained dentist to reduce technical variation.

***Supragingival plaque sampling*** Supragingival plaque samples from each subject were collected at Baseline (Day 0) and Day 21 following the procedures below. Subjects did not have oral hygiene practice include tooth brushing, flossing, mouth rinsing before sampling. Samples were collected after 2 hours food or drink (except water) intake. After MGI examination, each subject rinsed their mouth with 50ml sterilized water. After MGI examination 15 minutes, plaque along the gumline within 2 mm depth were collected with Gracey curette by qualified dentists. For each subject, plaque samples were collected for all teeth in two different quadrants (1 and 3 or 2 and 4) and pooled together in a single tube. Plaques on the Gracey curette were collected via swabbing with a sterilized cotton swab. The tips of swab were put into 0.6 ml TE20 buffer (20 mM Tris-HCl PH 8.0, 2 mM EDTA). Before isolating DNA, all samples were stored under -70°C.

***Plaque DNA extraction protocol*** Total DNA was extracted from Human Dental plaque following Dr. Larry Forney’s protocol with minor modifications ^7^. In general, frozen samples were thawed on ice before DNA isolation experiment. The original sample (250 ul) was transferred into a clean Bead-Beating-Tube (2ml Eppendorf tube). Sample suspensions were kept on ice while a Lytic-Enzyme Cocktail was prepared. Freshly prepared Lytic-Enzyme-Cocktail Master-Mix (100ul; containing 50 µl Lysozyme~500KU=10mg/ml, 6 µl Mutanolysin, 25 KU/ml, 3 µl Lysostaphin, 4000 U/ml in 20 mM sodium acetate and 41 µl TE buffer) was added to all samples and incubated at 37℃ for 45 min. To the lysate mix 750 mg cleaned and dry 0.1mm diameter Zirconia-Silica-Beads was added. Samples were subjected to bead beating for 2 minute at room temperature in a Qiagen TissueLyser LT (36 oscillations/second). One hundred and eighty µl of the crude lysate were transferred into a new tube and DNA isolated by Qiacube using DNeasy® Blood & Tissue Mini Kits.

***Bacterial 16S rRNA gene amplicon sequencing***. PCR amplicon libraries of 16S rRNA gene in the V1-V3 hyper-variable region were pyrosequenced as previously described ^6^. Totally, 182 plaque samples were obtained and analyzed from 91 individuals each of whom provided samples at Baseline and Day 21. Barcoded 16S rDNA amplicon sequencing using 454 Titanium yield a total of 1,419,998 processed reads (i.e., reads after quality assessment and control measures ^8^. The number of processed reads per sample ranged from 437 to 28, 456, with an average 7802 reads per sample (**Table S1**). All sequences were deposited at Sequence Read Archive under Accession ID SRA063171.

***Comparing the microbial structures of plaque microbiota***  Sequences were assigned to operational taxonomic units (OTUs) with a 97% threshold of pairwise identity, then classified using the Oral CORE reference database (CI 80%). For genus-level data, the function diversity in the “vegan” package of R was used to calculate alpha diversity indices. Genus-level PCA was performed in R using the “ade4” package ^9^ to visualize the difference of microbial community structure between different time points and treatments. Prior to this analysis, the data were sample-size normalized to decrease noise.

***Statistical analysis*** In this study, the significant difference for each bacterial taxon between CPC and placebo groups was established by Wilcoxon test and FDR correction. CCREPE ^10^ was employed to detect microbial interactions during CPC treatment at genus level. We applied this method to construct two bacterial interaction networks in CPC and control group. At Day21, pairwise Spearman correlations between all genera across all subjects within CPC or control group were first calculated. Finally, data was exported and visualized via Cytoscape ^11^ (<http://www.cytoscape.org>). In these networks, a node corresponds to the microbial abundance profile of a given microbe. Nodes are connected if they have a significant pairwise correlation across the environmental perturbations (*q*<0.1, FDR correction).

**Reference**

1. Loe H, Theilade E, Jensen SB. Experimental Gingivitis in Man. *J Periodontol* 1965 May-Jun; **36:** 177-187.

2. Offenbacher S, Barros SP, Paquette DW, Winston JL, Biesbrock AR, Thomason RG*, et al.* Gingival transcriptome patterns during induction and resolution of experimental gingivitis in humans. *J Periodontol* 2009 Dec; **80**(12)**:** 1963-1982.

3. Grant MM, Creese AJ, Barr G, Ling MR, Scott AE, Matthews JB*, et al.* Proteomic analysis of a noninvasive human model of acute inflammation and its resolution: the twenty-one day gingivitis model. *J Proteome Res* 2010 Sep 3; **9**(9)**:** 4732-4744.

4. Lee A, Ghaname CB, Braun TM, Sugai JV, Teles RP, Loesche WJ*, et al.* Bacterial and salivary biomarkers predict the gingival inflammatory profile. *J Periodontol* 2012 Jan; **83**(1)**:** 79-89.

5. Huang S, Li R, Zeng X, He T, Zhao H, Chang A*, et al.* Predictive modeling of gingivitis severity and susceptibility via oral microbiota. *ISME J* 2014 Mar 20.

6. Huang S, Yang F, Zeng X, Chen J, Li R, Wen T*, et al.* Preliminary characterization of the oral microbiota of Chinese adults with and without gingivitis. *BMC Oral Health* 2011; **11:** 33.

7. Ravel J, Gajer P, Abdo Z, Schneider GM, Koenig SS, McCulle SL*, et al.* Vaginal microbiome of reproductive-age women. *P Natl Acad Sci USA* 2011 Mar 15; **108 Suppl 1:** 4680-4687.

8. Schloss PD, Gevers D, Westcott SL. Reducing the effects of PCR amplification and sequencing artifacts on 16S rRNA-based studies. *PLoS One* 2011; **6**(12)**:** e27310.

9. Dray S, Dufour AB. The ade4 package: Implementing the duality diagram for ecologists. *J Stat Softw* 2007 Sep; **22**(4)**:** 1-20.

10. Faust K, Sathirapongsasuti JF, Izard J, Segata N, Gevers D, Raes J*, et al.* Microbial co-occurrence relationships in the human microbiome. *PLoS Comput Biol* 2012; **8**(7)**:** e1002606.

11. Shannon P, Markiel A, Ozier O, Baliga NS, Wang JT, Ramage D*, et al.* Cytoscape: a software environment for integrated models of biomolecular interaction networks. *Genome Res* 2003 Nov; **13**(11)**:** 2498-2504.
